# Induction immunosuppression and post-transplant diabetes mellitus: a propensity-matched cohort study

**DOI:** 10.3389/fendo.2023.1248940

**Published:** 2023-10-20

**Authors:** Suruchi K. Gupta, Elizabeth Mostofsky, Shweta R. Motiwala, Ali Hage, Murray A. Mittleman

**Affiliations:** ^1^ Department of Epidemiology, Harvard T.H. Chan School of Public Health, Boston, MA, United States; ^2^ Division of Endocrinology, Diabetes, and Metabolism, Department of Medicine, Beth Israel Deaconess Medical Center, Boston, MA, United States; ^3^ Harvard Medical School, Boston, MA, United States; ^4^ Division of Cardiovascular Medicine, Department of Medicine, Beth Israel Deaconess Medical Center, Boston, MA, United States; ^5^ London Health Sciences Centre, Schulich School of Medicine, Western University, London, ON, Canada

**Keywords:** induction, immunosuppression, diabetes, cardiac, transplant, heart, post-transplant diabetes mellitus

## Abstract

**Introduction:**

Post-transplant diabetes mellitus (PTDM) is a common complication among cardiac transplant recipients, causing diabetes-related complications and death. While certain maintenance immunosuppressive drugs increase PTDM risk, it is unclear whether induction immunosuppression can do the same. Therefore, we evaluated whether induction immunosuppression with IL-2 receptor antagonists, polyclonal anti-lymphocyte antibodies, or Alemtuzumab given in the peri-transplant period is associated with PTDM.

**Methods:**

We used the Scientific Registry of Transplant Recipients database to conduct a cohort study of US adults who received cardiac transplants between January 2008-December 2018. We excluded patients with prior or multiple organ transplants and those with a history of diabetes, resulting in 17,142 recipients. We created propensity-matched cohorts (n=7,412) using predictors of induction immunosuppression and examined the association between post-transplant diabetes and induction immunosuppression by estimating hazard ratios using Cox proportional-hazards models.

**Results:**

In the propensity-matched cohort, the average age was 52.5 (SD=13.2) years, 28.7% were female and 3,706 received induction immunosuppression. There were 867 incident cases of PTDM during 26,710 person-years of follow-up (32.5 cases/1,000 person-years). There was no association between induction immunosuppression and post-transplant diabetes (Hazard Ratio= 1.04, 95% confidence interval 0.91 – 1.19). Similarly, no associations were observed for each class of induction immunosuppression agents and post-transplant diabetes.

**Conclusion:**

The use of contemporary induction immunosuppression in cardiac transplant patients was not associated with post-transplant diabetes.

## Introduction

Post-transplant diabetes mellitus (PTDM) is a major cause of morbidity and mortality after solid organ transplant (1, 2). The risk of PTDM ranges from 10 - 40%, depending on the type of organ transplant, classification of diabetes, and type of agent (3). Higher rates of PTDM are seen following the use of maintenance immunosuppression agents such as calcineurin inhibitors, mTOR inhibitors (4), and high dose steroids (5, 6). Cardiac transplant recipients who develop PTDM are at higher risk of adverse outcomes including renal dysfunction, infection, and death (7–9).

In the peri-transplant period, over 50% of cardiac transplant recipients receive induction immunosuppression, with contemporary agents including interleukin-2 (IL-2) receptor antagonists, polyclonal anti-lymphocyte antibodies, and alemtuzumab (10). The goal of this treatment is to lower the risk of early rejection in high-risk patients, such as those who are younger, African American, highly sensitized, or are bridged to transplant using ventricular assist devices (11). However, the potential effects of this medication on PTDM remain unknown.

Studies evaluating the association between induction immunosuppression and PTDM in renal transplant recipients are inconclusive. Some studies suggest that the use of induction immunosuppression is associated with a lower rate of PTDM by enabling rapid corticosteroid withdrawal and lower doses of diabetogenic agents like tacrolimus (12, 13) while others have reported a higher rate of PTDM with the use of Basiliximab (14).

However, it is important to study these questions among cardiac transplant recipients rather than applying transplant guidelines that were developed based on data from kidney and liver transplant recipients (15, 16).

Therefore, we examined whether the use of any of the major categories of induction immunosuppression in the peri-transplant period is associated with clinically diagnosed post-transplant diabetes in a propensity-matched cohort study of patients who underwent cardiac transplantation in the United States between 2008 and 2018 with at least one year of follow-up. We also evaluated the associations between each of the three main categories of induction immunosuppression agents used in the contemporary era and the incidence of post-transplant diabetes.

## Methods

### Study population

This study used data from the Scientific Registry of Transplant Recipients (SRTR). The SRTR registry includes data on all donors, wait-listed candidates, and transplant recipients in the US, submitted by the members of the Organ Procurement and Transplantation Network (OPTN). The Health Resources and Services Administration (HRSA), U.S. Department of Health and Human Services provides oversight to the activities of the OPTN and SRTR contractors (17). The database includes detailed information on treatments received during the index hospitalization for each transplant. Follow-up data is recorded at six months and one year after transplant and annually thereafter. The protocol was reviewed by the Institutional Board Review at Harvard University and all data was protected per the university and SRTR standards.

We identified all adult patients aged 18 years or older who received their first heart transplant between January 1, 2008 and December 31, 2018, with at least one follow-up visit available as of August 2019. We excluded multiorgan transplant recipients and recipients with any organ transplant before their first heart transplant since these individuals may have previously received induction immunosuppression with multiple agents. This resulted in a total of 23,935 transplant recipients. We then excluded 6,793 recipients with diabetes diagnosed before their first heart transplant, resulting in a sample of 17,142 recipients available for analysis.

### Induction immunosuppression

We classified induction immunosuppression exposure based on medication data during the hospitalization for the first cardiac transplant on record for each patient. In our primary analysis, we classified participants as those who received induction immunosuppression and as those who did not. In secondary analyses, we examined the three major classes of induction agents used in the contemporary era: IL-2 receptor antagonists, polyclonal anti-lymphocyte antibodies, and alemtuzumab (18).

### Post-transplant diabetes

If a previously non-diabetic patient was diagnosed with new-onset diabetes at any time 6 months or later after the transplant, we classified them as an incident case of clinically diagnosed post-transplant diabetes. The diagnosis of diabetes was made by clinicians at the transplant center in the course of routine clinical care. The questionnaires include information about whether the patient was diagnosed with diabetes or not and whether it was insulin dependent or not (19). This data was reported to SRTR via completed questionnaires submitted every 6 months during the first year and annually thereafter.

### Statistical analysis

We created propensity scores using characteristics that, *a priori*, we expected to be predictors of receiving induction immunosuppression. We included the following characteristics in our propensity scores: recipient gender, age, body mass index (BMI), race/ethnicity, drug-treated hypertension, pre-transplant steroid use, life support status at the time of transplant, prior non-transplant cardiac surgery, ventricular assist device use before transplant, presence of hepatitis C virus (HCV) and cytomegalovirus (CMV) infection, presence of ischemic heart disease, history of cancer, intravenous antibiotics-treated infection while hospitalized for the index transplant, estimated glomerular filtration rate (MDRD equation) (20), history of any tobacco use, hospitalization status (home, inpatient, intensive care), functional status, use of cyclosporine, tacrolimus, mTOR inhibitors and steroids in the peri-transplant period, number of human leukocyte antigen mismatches, year of transplant (2008-2010, 2011-2013, 2014-2018), transplant center, and donor age. For variables with missing data, we created a separate category for missingness to reduce bias.

We used the psmatch2 command in Stata with a caliper width set to 0.001 to construct a cohort of patients prescribed induction immunosuppression matched 1:1 to patients who did not receive induction immunosuppression based on their propensity scores. We assessed the performance of the matching algorithm by calculating Student’s t-tests and Chi-square tests for continuous and categorical variables, respectively. We constructed Kaplan-Meier curves according to the use of induction immunosuppression in the peri-transplant period and estimated hazard ratios using Cox proportional-hazards regression models in the propensity matched cohorts. Similarly, we created propensity scores to analyze the association for each of the three major types of induction immunosuppression agents (IL-2 receptor antagonists alone, polyclonal anti-lymphocyte antibodies alone, and alemtuzumab alone), each compared to no induction immunosuppression.

In a sensitivity analysis, we reanalyzed the data in the full unmatched cohort using a multivariable Cox proportional hazards regression model adjusted for the same covariates that we had used to construct the propensity scores as described above.

Finally, to assess potential confounding by differences in induction immunosuppression therapy patterns across transplant centers, we repeated the analyses omitting transplant center from the propensity score model.

We tested for violations of the proportional hazards assumption by incorporating interactions between exposure and the natural logarithm of time and by examining the Schoenfeld residuals. All reported *p-*values were two-sided, and a p-value less than or equal to 0.05 was considered statistically significant. All analyses were performed using SAS 9.4 Statistical Software and Stata 16.1.

## Results

We identified 17,142 adult heart transplant recipients in the United States who received their first cardiac transplant between January 1^st^, 2008, and December 31^st^, 2018 who were free of diabetes mellitus and had not received a prior organ transplant. A total of 8,745 (51.0%) of these patients received induction immunosuppression following their first cardiac transplant. IL-2 receptor antagonists were the most commonly used class of induction immunosuppression, and basiliximab was the predominant IL-2 receptor antagonist used. Thymoglobulin was the predominant anti-lymphocyte polyclonal antibody used. After creating the propensity scores as described above, we matched 3,706 patients who received induction immunosuppression in the peri-transplant period to an equal number of cardiac transplant recipients who did not receive induction immunosuppression. **Table 1** shows differences in baseline characteristics between those treated and those not treated with induction immunosuppression in both the full cohort and in the propensity-matched cohort. After matching on propensity score, there were no statistically significant differences in the measured characteristics between the treatment groups.

**Table 1:** Characteristics of the study population by induction immunosuppression use, total (%) or Mean (SD), Scientific Registry of Transplant Recipients, 2008-2018.

		Full Cohort		Propensity Matched Cohort	
		Induction Therapy (n=8,745)	No Induction Therapy (n= 8,397)	Induction Therapy (n=3,706)	No Induction Therapy (n=3,706)
**Age (years)**		52.1 (13.5)	52.7 (13.2)	52.5 (13.2)	52.4 (13.4)
**Female**		2538 (29.0)	2208 (26.3)	1064 (28.7%)	1029 (27.8%)
Race/ethnicity					
	White	5828 (66.6)	5677 (67.6)	2483 (67.0%)	2498 (67.4%)
	Black	1963 (22.5)	1699 (20.2)	812 (21.9%)	780 (21.0%)
	Hispanic	620 (7.1)	661 (7.9)	96 (2.6%)	91 (2.5%)
	Asian	249 (2.9)	265 (3.2)	276 (7.4%)	294 (7.9%)
	Other	85 (1.0)	95 (1.1)	39 (1.1%)	43 (1.2%)
BMI (kg/m^2^)					
	Underweight (<18)	151 (1.7)	177 (2.1)	68 (1.8%)	64 (1.7%)
	Normal (18-24.9)	3265 (37.3)	3183 (37.9)	1381 (37.3%)	1420 (38.3%)
	Overweight (25-29.9)	3132 (35.8)	3020 (36.0)	1313 (35.4%)	1319 (35.6%)
	Obese (>30)	2168 (24.8)	2006 (23.9)	944 (25.5%)	903 (24.4%)
	Unknown	29 (0.3)	11 (0.1)		
**Past or current smoker**		3833 (43.8)	3826 (45.6)	1622 (43.8%)	1658 (44.7%)
**History of hypertension**	Yes	2439 (27.9)	2349 (28.0)	1033 (27.9%)	1010 (27.3%)
	No	2546 (29.1)	2315 (27.6)	1055 (28.5%)	1049 (28.3%)
	Unknown/Missing	3760 (43.0)	3733 (44.5)	1618 (43.7%)	1647 (44.4%)
**History of chronic steroid use in the past**	Yes	530 (6.1)	662 (7.9)	261 (7.0%)	251 (6.8%)
	No	8159 (93.3)	7612 (90.7)	3408 (92.0%)	3410 (92.0%)
	Unknown	56 (0.6)	123 (1.5)	37 (1.0%)	45 (1.2%)
**Prior ischemic heart disease**		2611 (29.9)	2667 (31.8)	1112 (30.0%)	1103 (29.8%)
**Prior cardiac surgery**	Yes	2248 (25.7)	2082 (24.8)	946 (25.5%)	939 (25.3%)
**Prior history of any malignancy**	Yes	738 (8.4)	634 (7.6)	305 (8.2%)	307 (8.3%)
**Life support use prior to transplant**		7159 (81.9)	6832 (81.4)	5810 (81.5)	5839 (81.9)
**eGFR (ml/min/1.73m^2^)**					
	≤30	485 (5.6)	263 (3.1)	173 (4.7%)	163 (4.4%)
	31 to <61	3485 (39.9)	2954 (35.2)	1446 (39.0%)	1444 (39.0%)
	61 to < 91	3316 (37.9)	3480 (41.4)	1434 (38.7%)	1438 (38.8%)
	91 to <125	1125 (12.9)	1296 (15.4)	500 (13.5%)	511 (13.8%)
	>=125	331 (3.8)	400 (4.8)	151 (4.1%)	148 (4.0%)
	Unknown	3 (0.03)	4 (0.1)	2 (0.1%)	2 (0.1%)
CMV status					
	Positive	4924 (56.3)	4603 (54.8)	2085 (56.3%)	2089 (56.4%)
	Negative	3531 (40.4)	3591 (42.8)	1552 (41.9%)	1548 (41.8%)
	Unknown	290 (3.3)	203 (2.4)	69 (1.9%)	69 (1.9%)
HCV status					
	Positive	177 (2.0)	171 (2.0)	76 (2.1%)	75 (2.0%)
	Negative	8285 (94.7)	7933 (94.5)	3520 (95.0%)	3512 (94.8%)
	Unknown	283 (3.2)	293 (3.5)	110 (3.0%)	119 (3.2%)
**VAD/TAH use**		2304 (26.4)	2491 (29.7)	975 (26.3%)	983 (26.5%)
HLA-DR mismatch					
	0	407 (4.7)	402 (4.8)	196 (5.3%)	183 (4.9%)
	1	3351 (38.3)	2975 (35.4)	1369 (36.9%)	1374 (37.1%)
	≥2	4485 (51.3)	3970 (47.3)	1877 (50.6%)	1883 (50.8%)
	Unknown	502 (5.7)	1050 (12.5)	264 (7.1%)	266 (7.2%)
HLA-AB mismatch					
	0	9 (0.1)	11 (0.1)	4 (0.1%)	5 (0.1%)
	1-3	1206 (13.8)	1083 (12.9)	497 (13.4%)	510 (13.8%)
	≥ 4	7027 (80.4)	6252 (74.5)	2941 (79.4%)	2925 (78.9%)
	Unknown	503 (5.8)	1051 (12.5)	264 (7.1%)	266 (7.2%)
**Steroids used at transplant**		8496 (97.2)	7946 (94.6)	3578 (96.5%)	3580 (96.6%)
Hospitalization status at transplant					
	Not Hospitalized	4747 (54.3)	4569 (54.4)	2013 (54.3%)	1974 (53.3%)
	ICU	2574 (29.4)	2451 (29.2)	1083 (29.2%)	1056 (28.5%)
	Hospitalized (Not in ICU)	1424 (16.3)	1377 (16.4)	610 (16.5%)	676 (18.2%)
Functional status					
	Hospitalized/Disabled	4403 (50.4)	4050 (48.2)	1816 (49.0%)	1802 (48.6%)
	Required assistance	2856 (32.7)	2693 (32.1)	1190 (32.1%)	1181 (31.9%)
	Normal/minor symptoms	1264 (14.5)	1262 (15.0)	535 (14.4%)	558 (15.1%)
	Missing	222 (2.5)	392 (4.7)	165 (4.5%)	165 (4.5%)
**Life support use at transplant**		7159 (81.9)	6832 (81.4)	3003 (81.0%)	3002 (81.0%)
Infection requiring IV antibiotics at transplant					
	Yes	839 (9.6)	826 (9.8)	336 (9.1%)	357 (9.6%)
	No	7816 (89.4)	7398 (88.1)	3318 (89.5%)	3300 (89.0%)
	Unknown	90 (1.0)	173 (2.1)	52 (1.4%)	49 (1.3%)
**Donor age (in years)**		31.6 (11.4)	32.0 (11.4)	32.2 (11.7)	32.0 (11.4)
Maintenance Immunosuppression in the peri-transplant period					
Tacrolimus		7868 (90.0)	7451 (88.7)	3336 (90.0%)	3332 (89.9%)
Cyclosporine		661 (7.6)	508 (6.1)	233 (6.3%)	237 (6.4%)
Mtor inhibitors		77 (0.9)	57 (0.7)	31 (0.8%)	31 (0.8%)

BMI, Body mass index; SD, Standard Deviation; eGFR, Estimated glomerular filtration rate; CMV, cytomegalovirus; HCV, Hepatitis C virus; VAD, Any ventricular assist device; TAH, Total artificial heart HLA, Human Leukocyte antigen; ICU, Intensive care unit; IV, Intravenous.

In the propensity-matched cohort, 867 patients developed post-transplant diabetes during 26,710 person-years of follow-up (32.5 cases/1,000 person-years). Alemtuzumab was associated with the lowest rate of post-transplant diabetes (**Table 2**).

**Table 2:** Rate of post-transplant diabetes mellitus by induction immunosuppression in propensity-matched cohorts*, Scientific Registry of Transplant Recipients, 2008-2018.

	No Induction therapy	Any Induction	Induction Agent		
			IL-2 receptor antagonists	Polyclonal anti-lymphocyte antibodies	Alemtuzumab
**n**	3,706	3,706	2,165	2,414	227
**Cases**	426	441	239	267	25
**Person-years**	13,363	13,347	7,992	8,309	1,358
**Rate per 1,000 person-years**	31.9	33	29.9	32.1	18.4

* Propensity score included: recipient gender, age, BMI, race, drug-treated hypertension, pre-transplant steroid use, life support status at the time of transplant, prior non-transplant cardiac surgery, ventricular assist device use before transplant, presence of hepatitis C and cytomegalovirus infection, presence of ischemic heart disease, history of cancer, IV antibiotics-treated infection while under hospitalization for index transplant, estimated glomerular filtration rate, history of any tobacco use, hospitalization status (home, inpatient, intensive care), functional status, steroid, tacrolimus, mtor inhibitor, cyclosporine use in the peri-transplant period, number of human leukocyte antigen mismatches, recipient center, year of transplant (2008-2010, 2011-2013, 2014-2018), and donor age.

Analyses of the propensity matched cohort (**Table 3**) shows that patients who received any induction immunosuppression had the same rate of post-transplant diabetes compared to those who did not (hazard ratio [HR] = 1.04, 95% confidence interval [CI] 0.91-1.19). Furthermore, when we examined the association according to the class of induction immunosuppression, there was no evidence of an association between any of the three classes of induction agents and the incidence of post-transplant diabetes compared to patients who did not receive any immunosuppression.

**Table 3:** Association between induction immunosuppression and post-transplant diabetes mellitus in full cohort and propensity-matched cohorts, Scientific Registry of Transplant Recipients, 2008-2018.

Exposure	Full Cohort			Propensity-Matched Cohort*		
	n	Hazard Ratio (95% CI)	P-Value	n	Hazard Ratio (95% CI)	P-Value
No Induction	8,397	1.00 (Reference)		3,706	1.00 (Reference)	
Any Induction	8,745	1.08 (0.96 – 1.21)	0.22	3,706	1.04 (0.91 – 1.19)	0.52
Induction Agent^#^						
No induction		1.00 (Reference)			1.00 (Reference)	
IL-2 receptor antagonists	4,595	1.03 (0.88 – 1.21)	0.73	2,165	1.10 (0.92 – 1.33)	0.30
Polyclonal anti-lymphocyte antibodies	3,673	1.19 (1.02 – 1.40)	0.02	2,414	0.98 (0.83 – 1.16)	0.82
Alemtuzumab	239	0.84 (0.38 – 1.87)	0.67	227	0.83 (0.47 – 1.5)	0.54

* Propensity score included: recipient gender, age, BMI, race, drug-treated hypertension, pre-transplant steroid use, life support status at the time of transplant, prior non-transplant cardiac surgery, ventricular assist device use before transplant, presence of hepatitis C and cytomegalovirus infection, presence of ischemic heart disease, history of cancer, IV antibiotics-treated infection while under hospitalization for index transplant, estimated glomerular filtration rate, history of any tobacco use, hospitalization status (home, inpatient, intensive care), functional status, steroid, tacrolimus, mtor inhibitor, cyclosporine use in the peri-transplant period, number of human leukocyte antigen mismatches, recipient center, year of transplant (2008-2010, 2011-2013, 2014-2018), and donor age.

# Each agent taken alone was compared with no induction therapy.

In analyses of the full cohort of 17,142 patients, we used multivariable Cox proportional hazards models adjusted for the same covariates used to construct the propensity scores. Results were similar to the results of the propensity-matched analyses for associations between any induction immunosuppression versus none (HR = 1.08, 95% CI: 0.96-1.21), and for associations of each of the three major classes of induction immunosuppression compared to no induction immunosuppression (**Table 3**).

**Figure 1:**
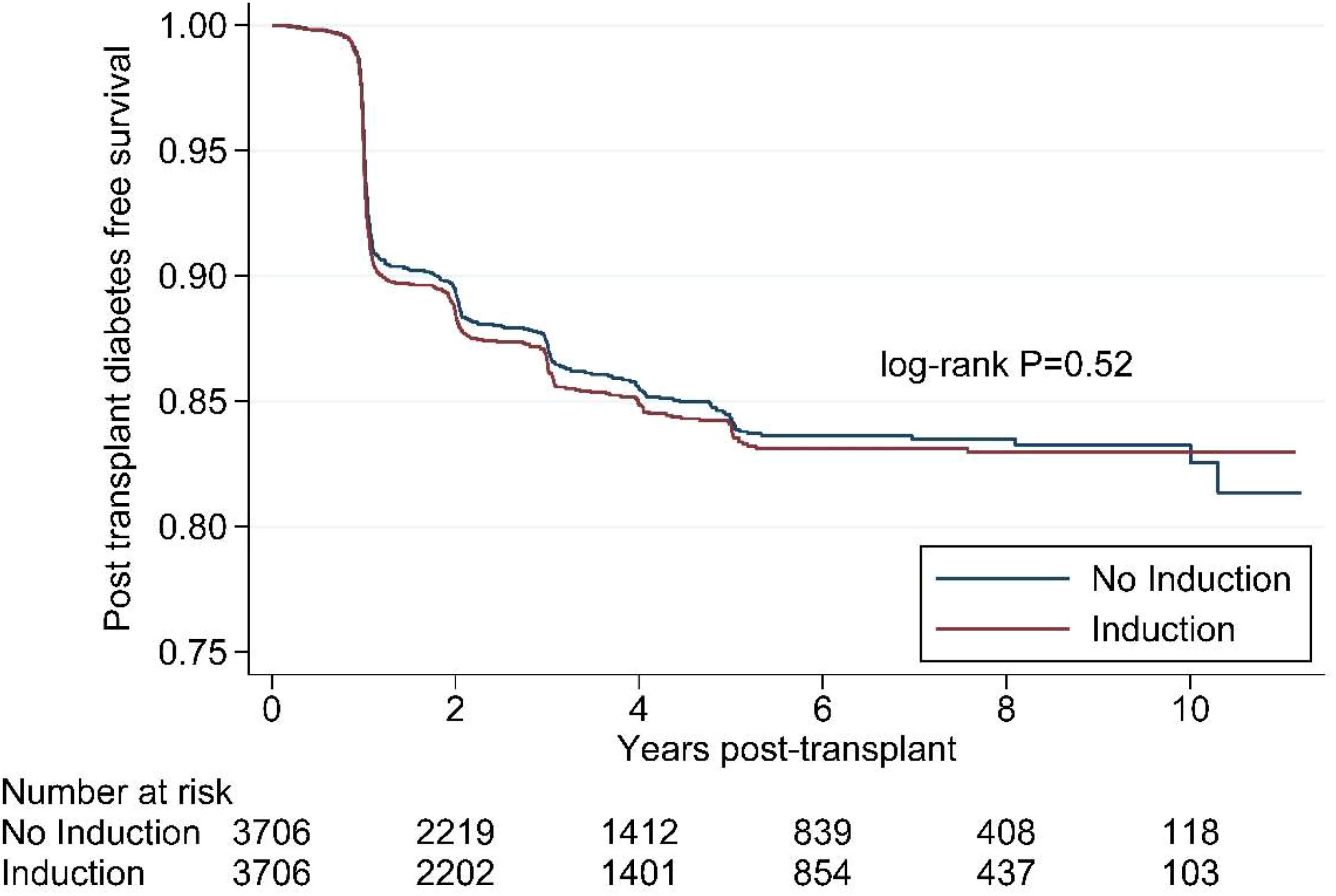
Kaplan Meier survival curve for induction immunosuppression and post- transplant diabetes in the propensity-matched cohort, Scientific Registry of Transplant Recipients, 2008-2018.

**Figure 1** shows the Kaplan Meier survival curves for the propensity matched cohort. The survival distributions for post-transplant diabetes mellitus over 10 years were similar between patients who did and did not receive any immunosuppression in the peri-transplant period (log-rank test: p=0.52).

In a sensitivity analysis that did not include treatment center in the propensity score model, compared to not receiving induction immunosuppression, treatment with any agent was associated with a 20% lower incidence of PTDM (HR=0.80, 95% CI: 0.73-0.88), suggesting strong confounding by differences in practice across treatment center.

## Discussion

In this contemporary cohort of cardiac transplant patients, approximately half of the patients (51%) received induction immunosuppression. The use of induction immunosuppression was not associated with the development of clinically diagnosed post-transplant diabetes. In our study, the strongest predictor of receiving induction immunosuppression was transplant center. When we did not account for the differences in practice pattern across centers, induction immunosuppression appeared to be associated with a lower rate of PTDM that was completely abolished after adjusting for confounding by differences across transplant centers.

We initially hypothesized that induction immunosuppression may lower the rate of post-transplant diabetes by lowering the risk of acute rejection (21, 22), reducing the need for high doses and long courses of steroids (13, 23, 24), and delaying the initiation of maintenance immunosuppressive agents such as cyclosporine and tacrolimus (25–27) that increase the risk of PTDM. However, after accounting for confounding by differences across transplant center, we observed no clinically meaningful association between induction immunosuppression and post-transplant diabetes.

To the best of our knowledge, no studies have examined the association between induction immunosuppression and post-transplant diabetes in cardiac transplant recipients. In a cohort of renal transplant patients, induction immunosuppression with basiliximab was associated with a higher risk of having an impaired glucose tolerance test 10 weeks post-transplant compared with no induction immunosuppression (15). However, patients in that study received prednisone 10 mg daily or higher. Since high-dose corticosteroids decrease insulin sensitivity and cause hyperglycemia, this finding may not reflect true PTDM (6).

Sharma and collegeaues (14) evaluated the association between basiliximab and the incidence of new-onset diabetes after transplant (NODAT), an earlier term for PTDM (28). After adjusting for age, BMI, HCV, CMV, acute rejection episodes within the first year, and maintenance immunosuppression agents such as tacrolimus and cyclosporine, the odds of developing NODAT was two times higher (odds ratio [OR] = 2.31, 95% CI: 1.37–3.88) in patients who received basiliximab (n=334) compared to those who did not. Bayes and colleagues (29) also reported that anti-CD 25 monoclonal antibodies were associated with a 3-fold higher odds (OR 3.28; 95% CI: 1.04 –10.31) of NODAT in a study of 74 renal allograft recipients. However, these are single-center studies with small sample sizes. Furthermore, the biological pathways by which basiliximab may cause PTDM are theoretical with no definitive evidence.

Similar to our findings, a meta-analysis of randomized control trials among renal transplant recipients (16) concluded that there was no difference in the incidence of PTDM among patients receiving alemtuzumab compared to those receiving anti-thymocyte globulin. However, the studies included in the meta-analysis did not compare induction immunosuppression to no induction therapy.

Some potential strengths and limitations of our study should be considered. This was a cohort study of over 17,000 cardiac transplant recipients receiving contemporary induction immunosuppression agents. All sites reported detailed information on recipient and donor demographics, clinical characteristics, and treatments. Furthermore, propensity matching on a robust set of potential confounders helped us address potential confounding by creating matched cohorts. Nonetheless, like any non-randomized study, there may be some residual or unmeasured confounding. A strength of the study is that diabetes was reported by the clinical team caring for the patients and reflected diagnoses that came to clinical attention during detailed annual clinical assessments. Furthermore, to minimize misclassification, we defined PTDM as newly diagnosed diabetes at least 6 months after transplant when most patients have been weaned from high dose corticosteroids that are sometimes used in the peri-transplant period (30, 31).

There is a large variation in practice and treatment with induction immunosuppression agents, especially in cardiac transplant recipients (26). As we observed in this cohort, contemporary literature suggests that only 50% of cardiac transplant recipients receive induction immunosuppression while close to 90% of renal transplant recipients receive some form of induction immunosuppression (10, 32). Use of induction immunosuppression in cardiac transplant recipients is only recommended in patients who are at an increased risk of acute rejection including black recipients and patients with HLA mismatches (11). Our analysis suggests that the clinical decision of whether or not to use induction immunosuppression should not be influenced by a patient’s underlying risk of developing PTDM.

Further research is warranted on the potential association between induction immunosuppression and other clinical outcomes such as infection, cancer, graft failure, and mortality. This can help cardiac transplant clinicians identify subgroups of patients who are most likely to benefit from the use of induction immunosuppression.

In conclusion, in this cohort study of cardiac transplant recipients, induction immunosuppression was not associated with the incidence of PTDM. These results imply that the use of induction immunosuppression among cardiac transplant recipients should be based on clinical indication and should not be influenced by a patient’s underlying risk for developing PTDM.

## Prior presentation

This work was presented in part at the American Heart Association’s Scientific Sessions, November 2020.

## Data availability statement

Publicly available datasets were analyzed in this study. This data can be found here: https://www.srtr.org/about-the-data/the-srtr-database/.

## Author contributions

SG: Primary Author: Conceived the presented idea, drafted the manuscript and data analysis. EM: Analysis and interpretation of the data, critical revision of the manuscript. SM: Expert opinion and critical revision of the manuscript. AH: Expert opinion and critical revision of the manuscript. MM: Acquisition, analysis and interpretation of data and critical revision of the manuscript. All authors contributed to the article and approved the submitted version.
